# Fatal Pelvic Hemorrhage Following the Use of the Aspirator Needle

**Published:** 1884-11

**Authors:** A. Reeves Jackson

**Affiliations:** Professor of Gynecology in the College of Physicians and Surgeons, of Chicago, etc.


THE CHICAGO
MEDICAL JOURNAL AND EXAMINER.

VOL. XLIX. NOVEMBER, 1884. No. 5.

### ORIGINAL COMMUNICATIONS.

ARTICLE I.

### FATAL PELVIC HEMORRHAGE FOLLOWING THE USE OF THE ASPIRATOR NEEDLE. *By* A. REEVES JACKSON, M. D., *Professor of Gynecology in the College of Physicians and Surgeons, of Chicago, etc.*

It is generally thought that the puncture made by the aspirating needle, whether for diagnostic or therapeutic purposes, is free from danger; and it is employed by most surgeons, without hesitation, both to determine the nature of doubtful swellings and to evacuate deep-seated abscesses, cystic collections, etc. The instrument has even been thrust into an aneurismal sac containing fluid blood without untoward consequences. It is true that death has followed its use in some cases in which it was employed to remove the fluid from ovarian cystomata, and these are the only ones, so far as I am aware, in which a fatal result has been attributed to it.

The following case shows, however, that the instrument is not so entirely safe as is usually supposed, and that even a fatal hemorrhage may follow its employment.

On August 16, I was called to see Mrs. L. E., in consultation, and received the following history: She was about 25 years of age, and had one living child three years old. During the summer of 1883 she had a premature labor, followed by septic fever, from which she perfectly recovered.

In the afternoon of August 8, after suppression of menstruation for three months, she was attacked with severe flooding, fainting, and labor-like pains. Dr. H., the medical attendant, was summoned, and found the os uteri dilated to the size of a quarter of a dollar. Later, in the evening, the hemorrhage and pains continuing, the vagina was tamponed. At midnight the tampon was removed, and the fetus escaped, leaving the placenta partially protruding from the os uteri. A portion of it was removed by traction, and as much as possible of the remainder was scraped away by means of a dull wire curette. August 10, the patient had a severe chill, followed by headache, fever, and uterine pain. The curette was again used, with the result of bringing away a few shreddy pieces, and the uterus was then injected with Churchill's solution of iodine. From that time onward there was a daily chill, with strongly-marked febrile symptoms, and continuous pelvic pain and tenderness.

On examination, I found general pelvic fullness with heat and great tenderness, especially in the right broad ligament. The uterus was bulky and slightly movable; the os sufficiently open to admit the end of the fingers. Issuing from the latter was an offensive, thin, bloody discharge. She was given morphine, potassic bromide, quinia, etc., and had intra-uterine injections of carbolized water. After two or three days the discharge lost its offensive character, but the inflammatory and febrile manifestations continued.

At this time, a brother of the patient, an accomplished physician, arrived, and took personal charge of her, so that every detail of treatment was assiduously enforced.

On the evening of the 22d I detected a soft spot in the right ligament, about one inch from the uterus. It was decided to introduce the aspirator needle at the place with the hope of finding pus. This was accordingly done under antiseptic precautions. There came into the receiving bottle about a dram and-a-half of serum, tinged with a little blood. Not feeling satisfied, I withdrew the needle, and re-introduced it, directing its point rather more toward the uterus, with the result of bringing away two or three drams of pure blood. The needle was then withdrawn, and the vagina syringed with hot carbolized water.

In a few moments the nurse informed me that there was bleeding from the vagina, and on returning to the patient, I found that there had indeed been a loss of two or three ounces of blood. I at once proceeded to tampon the vagina, and the hemorrhage appeared to be checked. As the patient was being lifted to a proper position in bed, after the operation, I observed a peculiar ashy hue overspreading her face, which I attributed to a partial fainting from the raising of her head.

Having an important engagement in the country, and barely time to catch the train, I was obliged, very reluctantly, to leave the patient at this critical juncture, in charge of her brother.

The latter informed me by letter, subsequently, that the blood, bright and arterial in character, to the extent of *two quarts*, continued to flow notwithstanding the presence of the tampon, which he tried to make firm, and that death ensued in less than an hour after the operation.

